# Antipsychotic Prescribing Trends for Autistic Adults with and without Intellectual Disability From 1997 to 2023: A Population-Based Cohort Study Using English Primary Care Records

**DOI:** 10.1192/bjo.2025.10218

**Published:** 2025-06-20

**Authors:** Aws Sadik, Antonio Pardiñas, Golam Khandaker, Dheeraj Rai, Paul Madley-Dowd

**Affiliations:** 1 Bristol Medical School, Bristol, United Kingdom; 2 MRC Integrative Epidemiology Unit, Bristol, United Kingdom; 3 Cardiff University School of Medicine, Cardiff, United Kingdom; 4 Centre for Neuropsychiatric Genetics and Genomics, Cardiff, United Kingdom; 5 Avon and Wiltshire Mental Health Partnership NHS Trust, Bristol, United Kingdom

## Abstract

**Aims:** Concern that antipsychotic over-prescribing has been harmful for autistic adults has led to deprescribing initiatives, including NHS England’s “Stopping the overmedication of people with ID, autism, or both” (STOMP) in 2015. It is unclear if there has been a subsequent change in prescribing rates or other possible aspects of over-prescribing. Thus, we sought to compare antipsychotic prescribing rates, recorded indications, mean doses and long-term use between autistic adults with and without intellectual disabilities (ID) and non-autistic adults in England from 1997 to 2023.

**Methods:** Using population-representative primary care records from the Clinical Practice Research Datalink Aurum, we identified adults 16–64 years old between 1997 and 2023 and stratified them into three groups: autistic adults with intellectual disability (ID), autistic adults without ID, and non-autistic adults. For each calendar year and group, we calculated (i) the proportion of adults prescribed an antipsychotic; (ii) the proportion of adults starting an antipsychotic; (iii) the proportion of first-time prescriptions with possible indications recorded 2 months either side; (iv) the mean daily doses for quetiapine, risperidone, aripiprazole and olanzapine; (v) the proportion of new courses that lasted over 1 year. We also performed analyses stratified by sex and age-group (16–29, 30–44, 45–64 years old).

**Results:** 45,143 autistic adults with ID, 121,071 autistic adults without ID, and 30,218,564 non-autistic adults entered the study. From 2001 to 2023 the percentage prescribed antipsychotics changed from 44% to 22% for autistic adults with ID, from 10% to 7% for autistic adults without ID, and from 1.1% to 1.2% for non-autistic adults. Over the same period, new prescription rates dropped from 5.2% to 1.6% for autistic adults with ID, from 2.0% to 1.1% for those without ID, and from 0.3% to 0.2% for non-autistic adults. Autistic adults with ID had consistently lower recording of possible indications: 40% in 2023 compared with 60% for the other two groups. They also had consistently higher proportions of courses lasting more than 1 year: 53% in 2022 compared with 43–44% in the other two groups. Mean doses prescribed were generally lowest for autistic adults with ID, followed by autistic adults without ID. Trends were largely similar across sex and age strata, with an exception being that prescribing rates have increased among autistic females without ID, from 6% in 2001 to 8% in 2023.

**Conclusion:** Disparities in antipsychotic prescribing rates in England have narrowed over the last 25 years, both before and after the 2015 launch of STOMP by NHS England.

